# Asymptomatic nasopharyngeal bacterial carriage, multi-drug resistance pattern and associated factors among primary school children at Debre Berhan town, North Shewa, Ethiopia

**DOI:** 10.1186/s12941-023-00557-3

**Published:** 2023-01-21

**Authors:** Chernet Belayhun, Mihret Tilahun, Abdurahaman Seid, Agumas Shibabaw, Bekele Sharew, Melaku Ashagrie Belete, Wondmagegn Demsiss

**Affiliations:** 1 Department of Medical Laboratory Science, Mehal Meda Hospital, North Showa, Ethiopia; 2 Department of Medical Laboratory Science, College of Medicine and Health Sciences, Wollo University, P.O. Box: 1145, Dessie, Ethiopia

**Keywords:** Antimicrobial susceptibility, Asymptomatic, Nasopharyngeal carriage, Primary school children

## Abstract

**Background:**

Nasopharyngeal carriage of bacteria is the main source for transmission of pathogens across individuals and horizontal spread of organisms in the community. It is an important risk factor for the acquisition of community-acquired respiratory tract infection. It is the major public health problem among children. The asymptomatic carriage of nasopharyngeal bacteria is different globally, particularly in Africa, carriage is higher in children and decreases with increasing age, 63.2% in children less than 5 years, 42.6% in children 5–15 years, and 28.0% in adults older than 15 years.

**Objective:**

The aims of this study was to determine asymptomatic nasopharyngeal bacterial carriage, multi-drug resistance pattern and associated factors among primary school children at Debre Berhan town, North Shewa, Ethiopia.

**Methods:**

Institutional based cross-sectional study was conducted at Debre Berhan town primary schools from February 1 to April 30, 2021. Primarily, the schools were stratified into two strata, public and private primary schools. From a total of sixteen government and fourteen private primary schools, five government and five private schools were selected by using a simple random sampling technique. Socio-demographic variables and potential risk factors were assessed using a structured questionnaire. A total of 384 nasopharyngeal swab samples were collected using sterile swabs aseptically; and inoculated on Blood agar, Chocolate agar, MacConkey agar, and Mannitol salt agar. The colony was characterized to isolate bacteria, and bacterial identification was performed by Gram reaction, hemolysis patterns, colonial characteristics and pigmentation, catalase test, coagulase test, mannitol fermentation test, oxidase test, fermentation of carbohydrates, H_2_S production, motility, formation of indole, triple sugar iron agar (TSI), citrate utilization, lysine decarboxylase or methyl red vogues proskur utilization, urea hydrolysis and satellitism tests. Antimicrobial sensitivity tests were performed by using modified Kirby-Bauer disk diffusion method. Data were entered into statistical package Epi data 4.0.0.6 and transferred to and analyzed using SPSS software version-23. P value of < 0.05 with Odds ratio (OR) and 95% confidence interval (CIs) was considered as statistically significant.

**Results:**

The overall prevalence of nasopharyngeal carriage of bacterial isolate was 35.7% (95% CI 30.7–40.7%). The predominant isolates were *Staphylococcus aureus* 54.5% followed by coagulase-negative *Staphylococcus* 35.8%, and *Streptococcus pyogens* 4.5%. Most bacterial isolates were susceptible to chloramphenicol, ciprofloxacin, gentamycin, nitrofurantoin, azithromycin, ciprofloxacin; and the overall multidrug resistance pattern of isolated bacteria was 62.03% out of 137 bacterial isolates. Numbers of rooms ≤ 2 per house [AOR = 5.88, 95%CI 1.26–27.57], having history of hospitalization [AOR = 4.08, 95%CI 1.45–11.53], passive smoking [AOR = 4.87, 95%CI 1.49–15.97], family size of > 5 members [AOR = 2.17, 95%CI 1.24–3.81], and number of students in the classroom [AOR = 2.35,95%CI 1.37–4.02] were statistically significant associated risk factors for nasopharyngeal bacteria carriage**.**

**Conclusion:**

Asymptomatic nasopharyngeal bacteria carriage in children is alarming for community-acquired infection. The overall multidrug resistance was very high. The risk of the carriage was increased with having a history of passive smoking, being in large family size and number of students per class. Longitudinal follow-up studies would be helpful for better understanding the infection risk in bacterial pathogen carriers.

## Introduction

An asymptomatic carrier is an individual or other living thing who has been infected with a microorganism without showing signs or symptoms. A carrier, whether they are not infected with the germ but the can transfer it to others or they develop symptoms later in the infection. Asymptomatic carriage of bacteria is a common phenomenon that occurs in the human nasopharynx including*, Streptococcus pneumoniae, Staphylococcus aureus, Haemophilus influenzae, Klebsiella pneumoniae,* and *Streptococcus pyogenes *[1, 2]*. S. aureus* and *S. pyogenes* have long been regarded as one of the most significant bacteria that cause disease in humans. They are the most common cause of abscess (boils), furuncle, and cellulite in the skin and soft tissues [3]. *Klebsiella pneumoniae* is also responsible for hospital-acquired urinary tract infections, pneumonia, septicemias, and soft tissue infections [4].

Monoclonal protein (M protein) and protein A is an essential virulence component discovered in *Streptococcus pyogenes and* *Staphylococcus aureus* cell walls (Pili) respectively. M protein exhibits antiphagocytic properties, as well as antigenically comparable epitopes to those identified in cardiac myosin and sarcolemma membrane proteins [5]. Capsular polysaccharides, lipopolysaccharides, fimbrial adhesins, and siderophores have all been linked to virulence in *K. pneumoniae* strains. The presence of the rmpA gene is linked to the hypervirulent phenotype of *K. pneumoniae* [6]. Potentially pathogenic bacteria in the nasopharynx of young children are responsible to cause otitis media, sinusitis, conjunctivitis, pneumonia, endocarditis, osteomyelitis, pyogenic arthritis, soft tissue infection, bacterial meningitis, sepsis, respiratory tract infections.

Pathogenic bacteria are more likely to colonize the nasopharynx of children and prone to recurrent otitis media, impaired local immunity, and exposure to respiratory diseases in various localities and enters the bloodstream, it can cause an invasive illness. Community nasal carriage s are widely different across the world, ranging from 3 to 25% [7, 8]. The nasopharyngeal carriage of *Streptococcus pneumoniae* and *Haemophilus influenzae* are mainly associated with the disease meningitis. The main reservoir of carriage and site of meningococcal spreading appears to be the upper respiratory tract [9]. *Streptococcus pyogens* cause a different spectrum of human infections, ranging from pharyngitis and pyoderma to life-threatening immunological complications such as rheumatic heart disease, post-streptococcal glomerulonephritis, toxic shock syndrome, and necrotizing fasciitis [10]. *Haemophilus influenzae* can survive inside respiratory epithelial cells; this intracellular sequestration may explain their capacity to colonize the respiratory epithelium for long periods of time [11]. *Staphylococcus aureus* is commonly found on the skin and in the nose of most healthy individuals and it is a leading cause of human bacterial infections [12].

Children are the target population group for developing pharyngitis, skin infection, as well as suppurative and non-suppurative complications [13]. Carriage is the first step in disease development and this allows the spreading of microorganisms within the community [14]. Antibiotic resistance is based on two major components: bacterial strains with suitable antibiotic resistance genes (acquisition of genes coding for resistance mechanisms and changes in housekeeping genes giving resistance) and antibiotic usage generating selective pressure. Resistance mutations mostly affect the drug target sites, whereas mobile genetic elements contain genes that are responsible for several kinds of resistance mechanisms [15].

Globally, particularly in Africa, the asymptomatic carriage of nasopharyngeal bacteria is different. Carriages are higher in children and decreases with increasing age, 63.2% in children less than 5 years, 42.6% in children 5–15 years, and 28.0% in adults older than 15 years [16].

Antimicrobial resistance (AMR) is a serious global threat to human, animal, and environmental health that is gaining traction. The emergence, spread, and persistence of multidrug-resistant (MDR) bacteria, also known as "superbugs," is to blame. MDR bacteria can be found in the animal, human, and environmental niches, and these pathogens are all linked in this triad [17–19]. Currently, lack of new antimicrobials on the horizon to replace ineffective drugs added urgency to the need to protect the efficacy of existing drugs [20, 21].

In industrialized countries and high-income populations, the prevalence of bacterial nasopharyngeal carriage in children is lower, and the average carriage prevalence usually settles at a lower level, around 20–50% [22–26]. In high-risk populations with a high burden of pneumococcal illness, the turnover and acquisition of novel strains is rapid, and pneumococcal carriage is common. Several factors, including environmental and socioeconomic factors, overcrowding conditions, and antibiotic overuse, appear to influence *S. pneumoniae* nasopharyngeal colonization, according to several studies [27–29].

The rise of antimicrobial resistance bacteria combined with the decreasing number of innovative antibacterial agents has led to warnings that we may soon lose our ability to treat bacterial infections [30]. Nasopharyngeal carriage by antimicrobial-resistant bacteria had been increasing in different parts of the world including Ethiopia [12] and previous study on preschool children on the study area had limitations on nasopharyngeal carriage [31], because of the absence of well-organized laboratories, inadequate distribution of vaccines, unavailability of antimicrobial agents, and lack of proper surveillance on the bacterial disease. Moreover, to the best of our knowledge, few studies were conducted in Ethiopia, they focused on patients and there is no much information on the nasopharyngeal bacterial carriage among school children in Debre Berhan town, Ethiopia. Therefore, the aimed of this study was to assess asymptomatic nasopharyngeal bacterial carriage, their multidrug resistance pattern and associated factors among primary school children at Debre Berhan town, North Shewa, Ethiopia.

## Method and materials

### Study design, area and period

An institution-based cross-sectional study was conducted from February 1 to April 30, 2021, at 10 primary schools in Debre Berhan town, North Shewa, Ethiopia. There are 16 government and 14 private primary schools with a total of 12,854 students. The allocated sample size were from Atse Zeryacobe 59 children, from Biruk Tesfa 93 children, from Model number two 57 children, from Selam Chora 5 children, from Adisketema 43 children, from Soresa 36 children, from Merit 11 children, from Abune Gorgoriwos 49 children, from new life 18 children, and from Abune Ephrem 13 children.

### Inclusion and exclusion criteria

All children who attend primary school and who were present at selected primary schools during the study period, were included in this study. On the other hand, children who were on antibiotics for the last two weeks and those with any signs and symptoms of respiratory diseases such as cough and watery nasal discharge at the time of data collection were excluded.

### Variables

The dependent variables were nasopharyngeal bacterial carriage and antimicrobial susceptibility profile of isolated bacteria. Whereas age, sex, grade level of children, school type, occupation of parents, educational status of parents, average monthly income, number of rooms in the house, bed-sharing with parents, passive smoking, family size, energy source for food cooking, history of hospitalization, number of students in the classroom were the independent variables.

### Operational definitions

- Asymptomatic carrier: apparently healthy individuals harboring the bacterial agent.
- Primary school: a school covering one up to eighth grades.
- Nasopharyngeal bacteria carriage: the presence of bacteria on the nasopharynx without causing symptomatic disease.
- Multidrug Resistance (MDR): is resistant to one or more antibiotics in three or more classes of antimicrobials agents [32].

### Sample size determination and sampling technique

The sample size was computed by using a single population proportion with assumption of (50%) prevalence.

$${\text{n}} = \frac{{{\text{z}}^{{2}} {\text{p}}({1} - {\text{p}})}}{{{\text{d}}^{{2}} }}$$

where: n = the minimum required sample size; z = Standard normal distribution value at 95% CI, which is 1.96, P = Expected prevalence of nasopharyngeal bacterial carriage in the study area, d = the margin of error taken as 5%. Since no studies were conducted in the study area, we took maximum proportion (p = 50%) and the sample size was calculated as follows.

$${\text{n}} = \frac{{{\text{z}}^{{2}} {\text{p}}(1 - {\text{p}})}}{{{\text{d}}^{{2}} }} = \frac{{(1.96)^{2} \,(0.5)\,(0.5)}}{{(0.5)^{2} }} = 384$$

A total of 384 school children were included in this study using stratified simple random sampling technique.

### Sampling technique

A stratified simple random sampling technique was used to select the study participants. Primarily, the schools were stratified into two strata, public and private primary schools. From a total of sixteen government and fourteen private primary schools, five government and five private schools were selected by using a simple random sampling technique. After that, the sample size was allocated to each selected primary school proportional to the number of students in each school. Similarly, the allocated number was distributed to each grade level proportionally. Finally, children were selected by using the list of student’s registration books as a sampling frame, a systematic random sampling method was employed to select the children from each grade. The total number of children in each grade was taken to calculate the sampling interval (K). The first child was randomly selected by lottery method and the next child was reached by every K^th^ interval. In case the selected child was not eligible, the next child was taken.

### Data, specimen collection and transportation

The questionnaire was prepared in English, then translated into the local language (Amharic), and re-translated back to English to keep the reliability of data collection. Socio-demographic data and other information were collected using structured questionnaires with a short interview guided by the principal investigator from guardians (family) of children. Demographic characteristics including age of children, sex, educational status of parents, occupation of parents, average monthly income, and associated factors like grade level of students, occupation of parents, bed-sharing with parents, passive smoking, family size, food cooking tools in the house, previous history of hospitalization and about single nasopharyngeal swab samples were collected by experienced and trained nurses from each study participant after appropriate instructions were given. The swab was placed in a sterile tube containing skim-milk tryptone glucose glycerol transport medium and transported to the Medical Microbiology Laboratory at Debre Berhan comprehensive specialized hospital. When unavoidable delay in processing was anticipated, nasopharyngeal swab samples were stored at 4 °C.

### Nasopharyngeal sample processing

The swab was gently introduced along the floor of the nasal cavity, passing under the inferior turbinate until it reached the pharyngeal wall, with the tip of the nose raised. The swab was removed carefully once it had made contact with the pharyngeal wall. The swab was placed in liquid transport media and kept refrigerated at 2–8 °C until it was transported on ice to microbiology laboratory [33, 34]. The nasopharyngeal swab was streaked onto Blood agar (HiMedia^™^), Chocolate agar, MacConkey agar (HiMedia^™^) and Mannitol salt agar (HiMedia^™^). The Chocolate agar was incubated in a candle jar at 37 °C for 24–48 h. Whereas, Blood Agar and Mannitol salt agar was aerobically incubated for 24 h at 37 °C. Positive growth on Blood agar and Mannitol salt agar (HiMedia^TM^) was subculture onto Nutrient agar (HiMedia^TM^) for biochemical and antimicrobial susceptibility tests [35, 36].

### Bacterial Isolation and identification

For biochemical and antimicrobial susceptibility test, positive growth on Blood agar and MacConkey agar (HiMedia™) were subcultured onto Nutrient agar (HiMedia™). The bacterial isolates were characterized using colony morphology, haemolysis pattern, Gram staining reaction; and through a panel of biochemical tests following the standard microbiological procedure. Gram-positive cocci were distinguished and recognized based on Gram stain, blood agar haemolysis patterns, colonial characteristics, catalase test, coagulase test, mannitol fermentation test and optochin (5 μg) susceptibility [33]. Gram-negative bacteria were identified based on Gram reaction, colony morphology (visual culture characteristics of a bacterial colony on an agar plate) and pigmentation, oxidase test, on triple sugar iron agar (TSI) fermentation of (glucose and lactose and H_2_S production), motility, formation of indole, and citrate utilization, lysine decarboxylase or methyl red vogues proskur utilization, urea hydrolysis and satellitism tests [36].

### Antimicrobial susceptibility testing

The isolated organisms were tested against antibiotic agents using the Kirby Bauer disc diffusion method on Muller Hilton agar (HMEDIA). Briefly, 3–5 pure colonies of isolated species from nutrient agar were picked and transferred to a tube containing 5 ml tryptone-soya broth and mixed to make a homogenous suspension, then incubated at 37 °C until the turbidity of the suspension was matched to a 0.5 McFarland standard. Using sterile swab, the inoculum suspension was inoculated over the entire surface of the Mueller Hinton agar plate. After application of the selected antimicrobial disks, the plate was incubated overnight at 37 °C for 16–18 h [37]. Antibacterial agents were selected based on local prescription habit and CLSI recommendations. The standard antibiotic discs (Liofilchem-Italy, HARDY Diagnosis-Santa Maria, USA) and their concentrations include: penicillin (10 μg), chloramphenicol (30 μg), ciprofloxacin (5 μg), clindamycin (30 μg), cefoxitin (30 μg), trimethoprim-sulfamethoxazole (1.25/23.75 μg), cefotaxime (30 μg), ceftriaxone (30 μg), erythromycin (15 μg) and oxacillin (30 μg) for Gram-positive bacteria; and chloramphenicol (30 μg), ciprofloxacin (5 μg), tetracycline (30 μg), gentamicin (10 μg), trimethoprim-sulfamethoxazole (1.25/23.75 μg), ceftriaxone (30 μg), piperacillin-tazobactam (100/10 μg), ceftazidime (30 μg), amikacin (30 μg), ampicillin (10ug), amoxicillin-clavulanic acid (20/10 μg), meropenem (10 μg) and amoxicillin (10ug) for Gram-negative bacteria [38, 39]. Inhibition zone diameter was measured and the degree of susceptibility was interpreted to each antibiotic according to CLSI guideline. Diameters of zones of inhibitions were measured using digital caliper. The interpretation of results of antimicrobial susceptibility tests were based on the standardized table supplied by CLSI [39] as sensitive, intermediate or resistant. Moreover, the isolate was considered multidrug resistant if non-susceptible to at least one agent in three or more antimicrobial categories, XDR was defined as non-susceptibility to at least one agent in all but two or fewer antimicrobial categories (i.e. bacterial isolates remain susceptible to only one or two categories was defined as non-susceptibility to agent in all [32].

### Quality assurance

Training was given for data collectors and completeness of the questionnaires was checked by principal investigator. Cultural outcome reliability was ensured by the implementation of standard quality control measures across the entire processes and standard operating procedures (SOPs) were followed. Quality control of culture media was verified for sterility test by overnight incubation of 5% one uninoculated plate/tube of the prepared media from each batch. Positive and negative controls were used for biochemical test media; and visual inspections of holes, uneven filling, and haemolysis, signs of freezing, bubbles and corrosion in media or plastic Petri dishes was conducted to check quality of all prepared culture media. Moreover, as supplied by CLSI standard reference strain of *S. aureus* (ATCC-25923), *E. coli* (ATCC-25922) and *P. aeruginosa* (ATCC-27853) were used for non- fastidious and for fastidious organisms *H. influenzae* (ATCC 49,247) and *S. pneumonia* (ATCC 49619) were used as quality control and for checking disk potency [39]. The supportive growth of Muller Hinton agar was checked by using *E. faecalis* (ATCC 29122 or 33186) and co–trimoxazole dis with the zone of inhibition of 20 mm or more in diameter.

### Statistical analysis

The data were entered every day into epi-data version 4.6.0.4. The data was then exported and analysed by using Statistical Package for Social Sciences (SPSS) version 25. The frequency and percentage descriptive statistics were calculated and presented using graphs and tables. In order to classify factors that spatially affect the frequency of dependent variables, bivariable analysis was computed. Variables with a P-value less or equal to 0.25 in bivariable analysis were subjected to multi-variable analysis. Adjusted odd ratio with p value of  < 0.05 with 95% CI was taken as statistically significant. Finally, the results were presented on text, graphs and tables.

## Results

### Sociodemographic characteristics of study participants

In this study, the age of study participants ranged from 7 to 14 years with a mean (± SD) age of 11.10 (± 2.11) years. Of the total 384 study participants, 66.9% study participants were from government schools and majority 60.2% of study participants were 11 to 14 years old. About half (51.6%) of the study participants were female. Two hundred fifty-eight (68.2%) of study participants were family members have two to four (Table 1).

**Table 1:** Socio-demographic characteristics of study participants (n = 384) in Debre Berhan town, North Shewa Ethiopia, February 1 to April 30, 2021

Variables	Categories	Frequency (%)
Sex	Male	186 (48.4)
	Female	198 (51.6)
Age	7–10	153 (39.8)
	11–14	231 (60.2)
Grade level	1–4	198 (51.6)
	5–8	186 (48.4)
School type	Government	257 (66.9)
	Private	127 (33.1)
Mother's education	Unable to read and write	44 (11.5)
	Primary	119 (31.0)
	Secondary	124 (32.3)
	Diploma /certificate	58 (15.1)
Father's education	Unable to read and write	29 (7.6)
	Primary	135 (35.2)
	Secondary	98 (25.5)
	Diploma/certificate	75 (19.5)
	Degree and above	47 (12.2)
Average monthly income	< 500 birrs	19 (4.9)
	501–1000 birr	47 (12.3)
	1001–1500 birr	179 (46.6)
	> 1501 birr	139 (36.2)
Mother's occupation status	Government	112 (29.2)
	Merchant	108 (28.1)
	Housewife	104 (27.1)
	Daily labor	52 (13.5)
	Others	8 (2.1)
Father's occupation status	Government	185 (48.2)
	Merchant	110 (28.6)
	Farmer	16 (4.2)
	Daily labor	67 (17.4)
	Others	6 (1.6)
No of room in the house	≤ 2	259 (67.4)
	3–4	102 (26.6)
	≥ 5	23 (6.0)
Family size	< 5	262 (68.2)
	≥ 5	122 (31.8)
Bed-sharing	No	245 (63.8)
	Yes	139 (36.2)
No of student in class	< 25	221 (57.6)
	≥ 25	163 (42.4)
Food cooking tools in house	Electricity	254 (66.4)
	Wood and charcoal	101 (26.3)
	Kerosine	29 (7.6)
Passive smoking	No	365 (95.1)
	Yes	19 (4.9)
History of hospitalization	No	358 (93.2)
	Yes	26 (6.8)

### Prevalence of bacterial infection and frequency of bacterial isolates

Among the total of 384 apparently healthy primary school children, the overall prevalence of bacterial infection was 35.7% (95% CI 30.7–40.7%), and the majority of bacteria carriage proportion was observed among children aged 7–10 years 58/153 (37.9%). About participant grade level, 94/137 (68.6%) of the isolates were isolated from low (1–4) grade level and about 99/137 (72.3%) isolates were from government school children. The proportion of nasopharyngeal bacteria isolated species in grade 1–4 is high which accounted 52.6% (72/137). Whereas, low proportion of Nasopharyngeal carriage was observed in grade eight which accounted 5.1% (Table 2).

**Table 2:** Frequency of bacterial isolates by grade level among asymptomatic primary school children in Debre Berhan town, North Shewa, Ethiopia, February 1 to April 30, 2021

Grade level of children									
Isolated bacteria species	Grade 1	Grade 2	Grade 3	Grade 4	Grade 5	Grade 6	Grade 7	Grade 8	Total (n/%)
*S. aureus* = *n (%)*	12 (16.4)	10 (13.7)	19 (26.07)	11 (15.1)	5 (6.8)	6 (8.2)	5 (6.8)	5 (6.8)	73 (53.3)
CoNS = n (%)	2 (10)	4 (20)	3 (15)	2 (20)	3 (15)	2 (10)	2 (10)	2 (10)	20 (14.6)
*S. pyogens* = *n (%)*	2 (20)	1 (10)	2 (20)	2 (20)	1 (10)	1 (10)	1 (10)	0 (0.0)	10 (7.3)
*K. pneumoniae* = *n (%)*	1 (10.)	1 (10)	3 (30)	1 (10)	2 (20)	1 (10)	(0.0)	1 (10)	10 (7.3)
*H. Influenzae* = *n (%)*	2 (22.2)	1 (11.1)	2 (22.2)	3 (33.3)	1 (11.1)	0 (0.0)	0 (0.0)	0 (0.0)	9 (6.6)
K. *rhinoscleromatis* = *n (%)*	0 (0.0)	2 (22.2)	1 (11.1)	2 (22.2)	3 (33.3)	1 (11.1)	2 (22.2)	0 (0.0)	9 (6.6)
*K. oxytoca* = *n (%)*	2 (33.3)	0 (0)	2 (33.3)	1 (16.7)	1 (16.7)	0 (0)	0 (0)	0 (0)	6 (4.4)
Total = n (%)	21 (15.3)	19 (13.9)	32 (23.4)	22 (16.1)	16 (11.7)	11 (8.02)	12 (9.0)	7 (5.1)	137 (100)

*CoNS* Coagulase negative *Staphylococcus*

### Phenotypic characteristics of the recovered isolates

**Fig. 1:**
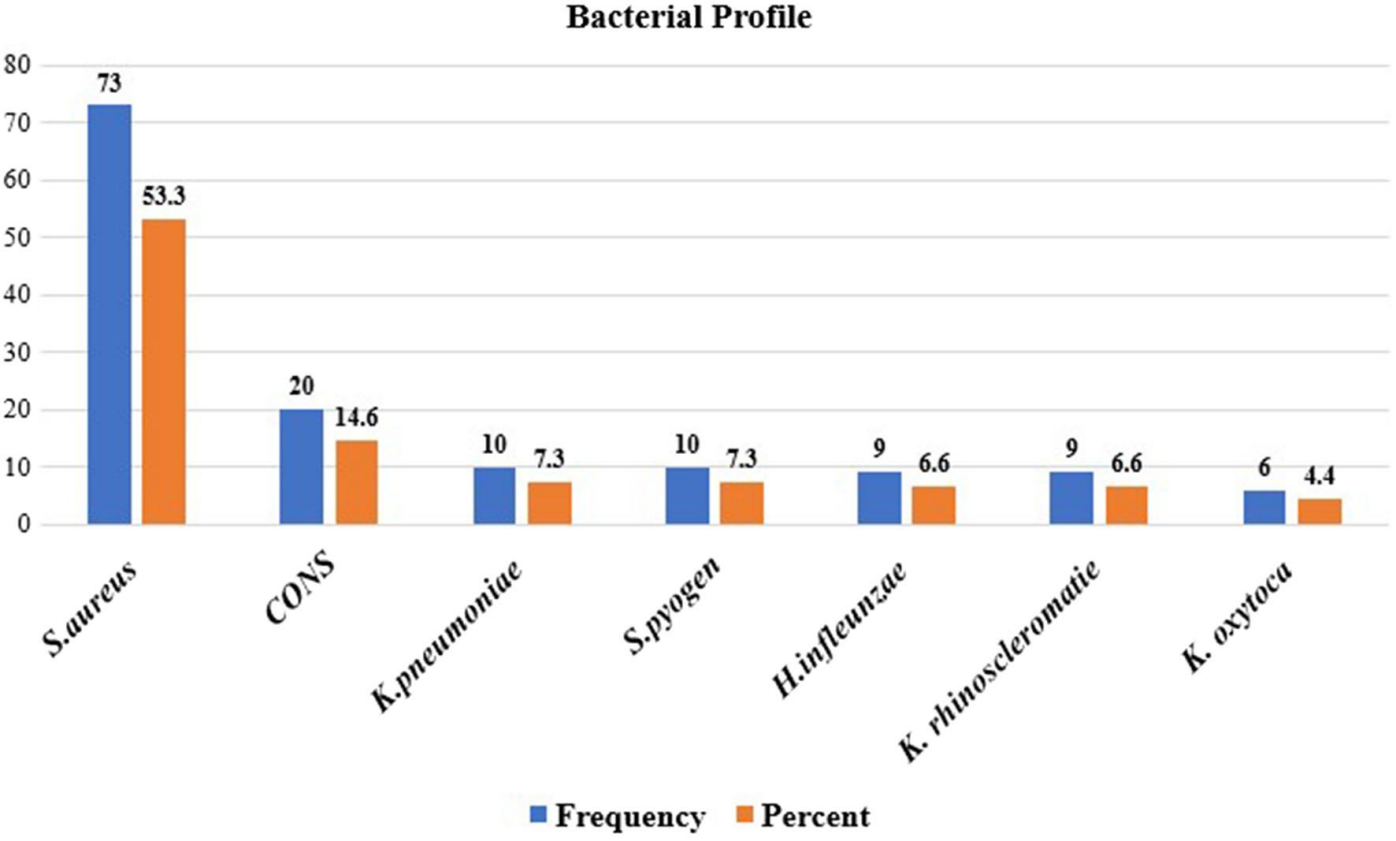
Distribution of bacterial species isolated among asymptomatic primary school children Debre Berhan town, North Shewa, Ethiopia, February 1 to April 30, 2021

Of the total 137 isolates, majority were Gram-positive bacteria which accounted 103 (75.2%). Overall, the predominantly isolated bacteria were *Staphylococcus aureus* 53.3% (73/137) followed by coagulase-negative *Staphylococcus* 14.6% (20/137), and *K. pneumoniae* 7.3% (10/137). However, the least bacterial isolates were *K. rhinoscleromatie* and *K. oxytoca* which accounts 996.4%) and 6 (44%) respectively (Fig. 1).

### Antimicrobial susceptibility profile of bacterial isolates

In general, Gram-positive bacteria showed resistance for tetracycline 87 (84.5%), trimethoprim-sulfamethoxazole 69 (74.2%), doxycycline 80 (86%), and chloramphenicol 70 (68%). Whereas, 57.3%, 64.1% and 51.5% of Gram-positive isolates were sensitive to clindamycin, azithromycin and erythromycin respectively. Moreover, about 79.5% of *S. aureus* isolates also showed resistance to methicillin (Table 3).

**Table 3:** Antimicrobial susceptibility profile of Gram-positive bacteria isolated from asymptomatic primary school children at Debre Berhan town, North Shewa Ethiopia, February 1 to April 30, 2021

Gram negative bacterial isolates		Antimicrobials tested										
		AZM N (%)	SXT N (%)	CLM N (%)	DOX N (%)	E N (%)	GM N (%)	CD N (%)	NIT N (%)	C N (%)	TET N (%)	FOX N (%)
*S. aureus* (73)	S	45 (61.6)	18 (24.7)	41 (56.2)	8 (11)	39 (53.4)	37 (50.7)	48 (65.8)	26 (31.8)	28 (38.4)	8 (11)	15 (20.5)
	R	28 (39.4)	55 (55.3)	32 (43.8)	65 (89)	34 (46.6)	36 (49.3)	25 (34.2)	49 (67.2)	45 (61.2)	65 (89)	58 (79.5)
CoNS (20)	S	12 (60)	6 (30)	13 (65)	5 (25)	11 (55)	13 (65)	11 (55)	5 (75)	5 (75)	2 (10)	–
	R	8 (40)	14 (70)	7 (35)	15 (75)	9 (45)	7 (35)	9 (45)	15 (75)	15 (75)	18 (90)	–
*S. pyogens* (10)	S	9 (900)	–	6 (60)	–	6 (66.7)	–	9 (90)	–	5 (66.7)	6 (60)	–
	R	1 (10.0)	–	4 (40)	–	4 (33.3)	–	1 (10.)	–	5 (33.3)	4 (40)	–
Total (103)	S	66 (64.1)	24 (40.4)	60 (57.3)	30 (31.9)	56 (51.5)	50 (53.2)	68 (65)	31 (33)	33 (32)	17 (15.5)	15 (20.5)
	R	38 (35.9)	69 (74.2)	44 (42.7)	80 (86)	50 (48.5)	44 (46.8)	36 (35)	63 (67)	70 (68)	87 (84.5)	58 (79.5)

*AZM* azithromycin, *CIP* ciprofloxacin, *CRO* ceftriaxone, *GM* gentamicin, *SXT* trimethoprim-Sulphamethoxazole, *CLM* clarithromycin, *AMP* ampicillin, *DOX* doxycycline, *E* erythromycin, *C* chloramphenicol, *TET* tetracycline, *FOX* cefoxitin, *NIT* nitrofurantoin, *CD* clindamycin

Most of the isolated Gram-negative bacteria showed resistance to tetracycline (94.1%), ampicillin (88.2%) and amoxacillin-clavulinic acid (76.5%). The resistance level of Gram-negative bacterial isolates against ceftazidime, tetracycline, trimethoprim-sulfamethoxazole, chloramphenicol, amoxacillin-clavulinic acid, cefotaxime, amikacin, ceftriaxone, gentamicin and ciprofloxacin ranged from 13 (38.2%) to 328 (94.1%). However, Gram-negative bacterial isolates were relatively sensitive against ciprofloxacin 21 (61.8%), amikacin 84 (55.9%) and meropenem 18 (52.9%) (Table 4).

**Table 4:** Antimicrobial susceptibility profile of Gram-negative bacteria isolated from the nasopharynx of asymptomatic primary school children at Debre Berhan town, North Shewa Ethiopia, February 1 to April 30, 2021

Gram negative bacterial isolates	Antimicrobials tested													
		C N (%)	TE N (%)	CIP N (%)	CXT N (%)	SXT N (%)	GN N (%)	AMP N (%)	AMC N (%)	CRO N (%)	CAZ N (%)	AMK N (%)	MEM N (%)	
*K. pneumoniae (*10)	S	3 (30)	1 (10)	6 (60)	4 (40)	3 (30)	6 (60)	1 (10)	2 (80)	4 (40)	5 (50)	6 (60)	5 (50)	
	R	7 (70)	9 (90)	4 (40)	6 (60)	7 (70)	4 (40)	9 (90)	8 (80)	6 (60)	5 (50)	4 (40)	5 (50)	
K. *rhinoscleromatie (*9)	S	2 (22.2)	1 (11.1)	5 (55.6)	5 (55.6)	2 (22.2)	4 (44.4)	1 (11.1)	3 (33.3)	5 (55.6)	5 (55.6)	4 (44.4)	6 (66.7)	
	R	7 (77.8)	8 (89.9)	4 (44.4)	4 (44.4)	7 (78.8)	5 (55.6)	8 (89.9)	6 (66.7)	4 (44.4)	4 (44.4)	5 (55.6)	3 (33.3)	
H. Influenzae (9)	S	3 (33.6)	0 (0)	6 (66.7)	5 (55.6	2 (22.2)	5 (55.6)	1 (11.1)	2 (22.2)	5 (55.6	3 (33.3)	5 (55.6)	6 (66.7)	
	R	6 (66.4)	9 (100)	3 (33.3)	4 (44.4)	7 (77.8)	4 (44.4)	8 (88.9)	7 (77.8)	4 (44.4)	6 (66.7)	4 (44.4)	3 (33.3)	
*K. *oxytoca (6)	S	1 (29)	0 (0)	4 (80)	3 (50)	2 (33.3)	3 (50)	2 (33.3)	1 (16.7)	4 (66.7)	3 (50)	4 (66.7)	2 (33.3)	
	R	5 (71)	6 (100)	2 (20)	3 (50)	4 (66.7)	3 (50)	4 (66.7)	5 (83.3)	2 (33.3)	3 (50)	2 (33.3)	4 (66.7)	
Total (34)	S	9 (26.5.4)	2 (5.9)	21 (61.8)	17 (50)	9 (26.5)	18 (52.9)	4 (11.8)	8 (23.5)	18 (52.8)	16 (47)	19 (55.9)	18 (52.9)	
	R	25 (73.5)	32 (94.1)	13 (38.2)	17 (50)	25 (73.5)	17 (47.1)	31 (88.2)	26 (76.5)	17 (47.1)	18 (53)	15 (44.1)	16 (47.)	

*NT* note tested, *AMP * Ampicillin, *GN* Gentamicin, *AMK*  Amikacin *CIP*  Ciprofloxacin (: 5 μg), *SXT* trimethoprim-sulfamethoxazole, MEM meropenem, *AMC*  Amoxicillin-clavulanic acid, *CTX*  cefotaxime, *CAZ*  ceftazidime, *CRO*  ceftriaxone, *TE* Tetracycline (:30 μg) C  Chloramphenicol, *TZP* piperacillin tazobactam, *R*  resistant, *S*  sensitive

### Multiple drug resistance (MDR) Profile of the isolates

Overall, 125 (91.12%) bacterial isolates were resistant to at least one antimicrobial agent and 109 (79.6%) isolates were resistant to ≥ 2 antimicrobials agents. Thirty-six (26.3%) isolates had developed resistance to five and more than five antimicrobials. The overall Multidrug resistance (MDR = non-susceptible to ≥ 1 agent in ≥ 3 antimicrobial classes) of isolated bacteria was 62.03% and there was no PDR. The MDR of Gram-negative and Gram-positive isolates was 56.1% and 55.9%, respectively. About 68.3% of *S. aureus,* 66.7% of *H. influenzae* and K. *rhinoscleromatis,* 60% of *CONS* and *K. pneumoniae*, 40% of *S. pyogen,* and 16.7% of *K. oxytoca* isolates developed MDR (Table 5).

**Table 5:** Multiple drug resistance patterns of bacteria isolated from the nasopharynx of asymptomatic primary school children at Debre Berhan town, North Shewa Ethiopia, February 1 to April 30, 2021

Isolated bacteria	Antimicrobial resistance pattern							MDR n ≥ 3
	R_o;_ n (%)	R_1_ n (%)	R_2_ n (%)	R_3_ n (%)	R_4_ n (%)	R_5_ n (%)	≥ R_6_	
Gram positive	7 (6.8)	12 (11.7))	18 (17.5)	19 (18.4)	17 (16.5)	20 (19.4)	10 (9.7)	66 (56.1)
*S. aureus (73)*	2 (2.7)	9 (12.3)	12 (14.4)	14 (19.2)	11 (15.1)	15 (20.5)	10 (13.7)	50 (68.5)
*CONS (*20)	3 (15)	2 (10)	3 (15)	4 (20)	5 (25)	3 (15)	–	12 (60)
*S. pyogens (*10)	2 (20)	1 (10)	3 (30)	1 (10)	1 (10)	2 (20)	–	4 (40)
Gram negative	4 (11.8)	4 (11.8)	6 (17.6)	7 (20.6)	6 (17.6)	6 (17.6)		19 (55.9)
*K. pneumoniae (*10)	1 (10)	1 (10)	2 (20)	2 (20)	2 (20)	2 (20)		6 (60)
*H. influenzae (*9)	1 (11.11)	1 (11.11)	1 (11.11)	2 (22.2)	2 (22.2)	2 (22.2)		6 (66.7)
K. *rhinoscleromatis (*9)	1 (11.11)	1 (11.11)	1 (11.11)	3 (33.33)	1 (11.11)	2 (22.2)		6 (66.7)
*K. oxytoca (*6)	1 (16.7)	1 (16.7)	2 (33.33)	–	1 (16.7)	–		1 (16.7
Overall Total^b^	11 (8.02)	16 (11.7)	24 (17.5)	26 (18.98)	23 (16.8)	26 (18.98)	10 (7.3)	85 (62.04%)

*MDR* multi-drug resistant, *R0* No antibiotic resistance, *R1* resistance to one class, *R2* resistance to two class, *R3* resistance to three class, *R4* resistance to four class, *R4* resistance to four antibiotics class, *R5* resistance to five and more than five antibiotics class

^a^Percent is computed from the total number of each bacteria species

^b^Percent is computed from a total number of isolates

### Factors associated with nasopharyngeal bacteria carriage

In the current study, bivariable analysis was performed and sociodemographic characteristics such as sex, age, average monthly income, and food cooking tools in the house were not significantly associated with nasopharyngeal bacteria carriage.

All variables with a P-value less or equal to 0.25 in bivariable analysis were subjected to multi-variable analysis and the absence of multicollinearity and goodness of fit for The Hosmer–Lemeshow test were entered into multivariable logistic regression analysis. Age, grade level, school type, education, occupation status of parents, average monthly income, and bedsharing were not significantly associated with nasopharyngeal bacteria carriage. However, family size living together > 5 members [AOR = 2.17, 95%CI 1.24–3.81, P = 0.006], number of rooms ≤ 2 per house [AOR = 5.88, 95%CI 1.26–27.57, P = 0.024], having history of hospitalization [AOR = 4.08, 95%CI 1.45–11.53, P = 0.008], passive smoking [AOR = 4.87, 95%CI 1.49–15.97, P = 0.009], and number of students > 25 in the classroom [AOR = 2.35,95%CI 1.37–4.02, P = 0.002] were statistically significant associated risk factors for nasopharyngeal bacteria carriage (Table 6).

**Table 6:** Factors associated with nasopharyngeal bacteria carriage among asymptomatic primary school children using bivariate and multivariate analysis at Debre Berhan town, North Shewa Ethiopia, February 1 to April 30, 2021

Variables	Total (n/%)	Nasopharyngeal bacteria carriage		COR (95% CI)	P-value	AOR (95%CI)	P-value
		Yes (n/%)	No (n/%)				
Sex							
Male	186 (48.4)	63 (33.9)	123 (66.1)	1.05 (0.69–1.60)	0.829	–	
Female	198 (51.6)	65 (32.8)	133 (67.2)	1			
Age							
7–10	153 (39.8)	58 (37.9)	95 (62.1)	1.40 (0.91–2.16)	0.122	1.17 (0.53–2.60)	0.696
11–14	231 (60.2)	70 (30.3)	161 (69.7)	1		1	
Grade level							
1–4	198 (51.6)	76 (38.4)	122 (61.6)	1.60 (1.05–2.47)	0.031	1.67 (0.75–3.75)	0.206
5–8	186 (48.4)	52 (28.0)	134 (72.0)	1		1	
School type							
Government	257 (66.9)	99 (38.5)	158 (61.5)	2.12 (1.30–3.44)	0.002	1.37 (0.73–2.57)	0.324
Private	127 (33.1)	29 (22.8)	98 (77.2)	1		1	
Mother’s education							
Unable to read and write	44 (11.5)	23 (52.3)	21 (47.7)	6.02 (2.10–17.3)	0.010	4.1 (1.00–15.39)	0.056
Primary	119 (31.0)	45 (37.8)	74 (62.2)	3.35 (1.23–8.61)	0.012	2.43 (0.77–7.64)	0.129
Secondary	124 (32.3)	36 (29.0)	88 (71.0)	2.25 (0.87–5.83)	0.950	1.86 (0.60–5.57)	0.281
Diploma /certificate	58 (15.1)	18 (31.0)	40 (69.0)	2.47 (0.88–6.95)	0.850	2.96 (0.90–9.75)	0.073
Degree and above	39 (10.2)	6 (15.4)	33 (84.6)	1		1	
Father’s education							
Unable to read and write	29 (7.6)	18 (62.1)	11 (37.9)	4.77 (1.76–12.93)	0.020	1.64 (0.44–6.13)	0.466
Primary	135 (35.2)	47 (34.8)	88 (65.2)	1.56 (0.74–3.28)	0.244	0.82 (0.32–2.09)	0.678
Secondary	98 (25.5)	35 (35.7)	63 (64.3)	1.62 (0.75–3.52)	0.222	0.89 (0.35–2.29)	0.819
Diploma/certificate	75 (19.5)	16 (21.3)	59 (78.7)	0.79 (0.34–1.86)	0.592	0.56 (0.21–1.49)	0.250
Degree and above	47 (12.2)	12 (25.5)	35 (74.5)	1		1	
Average monthly income						–	
< 500 birrs	19 (4.9)	9 (47.4)	10 (52.6)	2.22 (0.84–5.89)	0.107	1.28 (0.35–4.57)	0.706
501–1000 birr	47 (12.2)	19 (40.4)	28 (59.6)	1.68 (0.84–3.34)	0.140	1.85 (0.78–4.34)	0.159
1001–1500 birr	179 (46.6)	60 (33.5)	119 (66.5)	1.25 (0.77–2.02)	0.367	1.30 (0.74–2.31)	0.364
> 1501 birr	139 (36.2)	40 (28.8)	99 (71.2)	1		1	
Mother’s occupational status							
Government	112 (29.2)	27 (24.1)	85 (75.9)	1		1	
Merchant	108 (28.1)	39 (36.1)	69 (63.9)	1.78 (0.99–3.19)	0.053	1.57 (0.76–3.26)	0.217
Housewife	104 (27.1)	40 (38.5)	64 (61.5)	1.97 (1.09–3.54)	0.024	1.10 (0.52–2.34)	0.794
Daily labor	52 (13.5)	20 (38.5)	32 (61.5)	1.97 (0.97–3.99)	0.061	0.94 (0.39–2.40)	0.898
Father’s occupational status							
Government	185 (48.2)	49 (26.5)	136 (73.5)	1		1	
Merchant	110 (28.6)	44 (40.0)	66 (60.0)	1.85 (1.12–3.06)	0.016	1.13 (0.58–2.18)	0.710
Farmer	16 (4.2)	8 (50.0)	8 (50.0)	2.70 (0.99–7.79)	0.053	1.45 (0.39–5.38)	0.574
Daily labor	67 (17.4)	25 (37.3)	42 (62.7)	1.65 (0.91–2.99)	0.097	0.71 (0.32–1.61)	0.418
Others	6 (1.6)	2 (33.3)	4 (66.7)	1.38 (0.25–7.82)	0.710	0.82 (0.10–6.86)	0.862
No of room in the house							
< 2	259 (67.4)	99 (38.2)	160 (61.8)	6.49 (1.49–28.3)	0.013	5.88 (1.26–27.57)	0.024**
3–4	102 (26.6)	27 (26.5)	75 (73.5)	3.78 (0.83–17.21)	0.086	2.79 (0.56–13.98)	0.211
> 5	23 (6.0)	2 (8.7)	21 (91.3)	1		1	
Family size							
< 5	262 (68.2)	74 (28.2)	188 (71.8)	1		1	
> 5	122 (31.8)	54 (44.3)	68 (55.7)	2.02 (1.29–3.15)	0.002	2.17 (1.24–3.81)	0.006**
Bed-sharing							
No	245 (63.8)	67 (27.3)	178 (72.7)	1		1	
Yes	139 (36.2)	61 (43.9)	78 (56.1)	2.08 (1.34–3.22)	0.010	1.54 (0.90–2.65)	0.113
No of students in class							
≤ 25	221 (57.6)	53 (24.0)	168 (76.0)	1		1	
> 25	163 (42.4)	75 (46.0)	88 (54.0)	2.70 (1.75–4.18)	p ≤ 0.01	2.35 (1.37–4.02)	0.002**
Food cooking tools in house						–	
Electricity	254 (66.4)	84 (33.1)	170 (66.9)	1			
Wood and charcoal	101 (26.3)	37 (36.6)	64 (63.4)	1.17 (0.72–1.89)	0.523		
Kerosine	29 (7.6)	7 (24.1)	22 (75.9)	0.64 (0.26–1.57)	0.332		
Passive smoking							
No	365 (95.1)	115 (31.5)	250 (68.5)	1		1	
Yes	19 (4.9)	13 (68.4)	6 (31.6)	4.71 (1.75–12.70)	0.020	4.87 (1.49–15.97)	0.009**
History of hospitalization							
No	358 (93.2)	111 (31.0)	247 (69.0)	1		1	
Yes	26 (6.8)	17 (65.4)	9 (34.6)	4.20 (1.82–9.72)	0.010	4.08 (1.45–11.53)	0.008**

*AOR* adjusted odds ratio, *CI* confidence interval, *COR* crude odds ratio, *Ref* reference

^*^*Significant at p < 0.05

## Discussion

Asymptomatically, the human nasopharynx contained a diverse range of microorganisms, ranging from commensal bacteria to potential pathogens [40].

In the present study, the overall phenotypic/culture positivity of asymptomatic nasopharyngeal bacteria carriage of school children was 35.7% (95% CI 30.7–40.7%). This is comparable with previous study done in Debreberhan Ethiopia 29.9 % and 36.7% [31, 41], Istanbul 31.2% [42] and Tanzania 23.5% [43]. However, it was lower than previous reports in Czech Republic 62.8% [14], Korea 53.9 % [44] and in Jimma, Ethiopia 47.74% [45]. The observed inconsistency might be due to methodological differences, sample size and seasonal variations, sociodemographic variability of the study participants.

In the present study, *Staphylococcus aureus* 53.3% (73/137) followed by *coagulase-negative Staphylococcus* 14.6% (20/137*), and K. pneumoniae* 7.3% (10/137). The Predominate bacteria on this study is comparable with the study in reported in Jimma Ethiopia 52.26% [46] and Nigeria 56.3% [47]. However, the current result was higher than a studies reported from Ghana [48], Korea 18.2% [44], Nepal 16.6 [49]. Indonesia 7.3% [50], Istanbul 7.9% [42]. These variations might be due to sociodemographic and economic characteristics of the population under study and exposure to different potential risk factors. Additionally, Person-to-person contact or contact with an infected object, such as a doorknob, is increases the bacteria spreads. *Staphylococcus aureus* to hang around in your nasal passages, therefore a staph infection is prevalent in the nose [51].

In our study, the carriage of coagulase-negative *Staphylococcus* was 14.6 %, which is lower than findings from Ghana 47.3% [52], and Bahirdar Ethiopia 12% [53]. In the same way, the finding of *Klebsiella* species carriage was greater than 4.4 %, which is lower than the finding reported from Indonesia 11% [54]. In our study, the prevalence of *Streptococcus pyogens* was *7.3* %. This finding was higher than study done in Istanbul 2.9% [42], Nepal 5.3% [49], and lower than that of the previous report in Hawassa Ethiopia 12.2% [55]. Similarly, the prevalence of *Haemophilus influenzae* isolates was 6.6 %, which is higher than the finding in German 0.3% [56]. However, our finding was lower than the reports in Indian 27.5% [57], Mediterranean coast region 27.7% [42], Korea 13.6% [44], the Czech Republic 24.9% [14], Nepal 3.4% [49], and Istanbul 11.2% [42]. The carriage of these bacteria appears to fluctuate in a dynamic process throughout the host lifetime. This wide variation might be attributable to discrepancies in the study demographic conditions as well the sample size of the study participants.

According to the international standard for the definition of drug resistance [32], 62.04 % of the total bacterial isolates showed resistance to more than three classes or categories of antibiotics (MDR). The reason for high MDR prevalence might be due antimicrobial drugs are freely and widespread availability in the community in most underdeveloped nations, including Ethiopia, and it is normal practice to buy antimicrobials without a prescription. The rising of resistance to these antimicrobial drugs could be linked to their broad usage in the context for the treatment of diverse illnesses due to their ease of administration, relative cost effectiveness and poor infection control stgies, inappropriate utilization of antimicrobial agents in empirical treatment, extreme antibiotic use and self-antibiotics prescribing habits [58].

Regarding the antimicrobial susceptibility pattern of isolated bacteria, our study revealed that *Staphylococcus aureus* isolates were resistance to trimethoprim-sulfamethoxazole (55.3%), doxycycline (89%) and chloramphenicol (61.2%). The resistance to these antibiotics was higher than previous studies from Jordan; trimethoprim-sulphamethaxazole (20%), and doxycycline (13.4% [59]. A similar resistance level was seen from previous study in Debreberhan Ethiopia [41]. This might be due to drug uptake limitation, drug target modification, drug enzymatic inactivation, and active efflux of the drug and drug buildup is reduced by genetic changes that change the target DNA gyrase or diminish outer membrane proteins [60, 61]. *Staphylococcus aureus* isolates were susceptible (61.6%) to azithromycin, (53.4%) to erythromycin and (65.8%) to clindamycin.

From a total of 73 *Staphylococcus aureus* isolates 79.5% was methicillin resistant. The prevalence of methicillin resistant *Staphylococcus aureus* on the current study was higher than previous studies which were reported in Iran 35.9% [62], Pokhara, Nepal 56.1% [63], and Ethiopia, Bahir Dar 13.8 % [64], Jimma 18.8% [45], and contraindicated to study reported at Debre Markos (all the isolates were sensitive to cefoxitin, mean there was no MRSA isolate [65]. *Staphylococcus aureus* develops antimicrobial resistance through a variety of mechanisms. Limiting drug uptake, modifying the drug target, enzymatic inactivation of the drug, and active efflux of the drug are some of these methods. In addition to these, resistance to methicillin is caused by the *mec*A gene which codes the low affinity penicillin-binding protein (PBP2a) or (PBP 2'). β-lactam antibiotic normally binds to PBPs in the cell wall, resulting in the disruption of synthesis of the peptidoglycan layer and death of the bacterium. The mecA gene is primarily responsible for MRSA resistance. PBP-2a, a novel penicillin-binding protein, is encoded by the mecA gene. Anti-staphylococcal drugs, such as methicillin, flucloxacillin, dicloxacillin, and nafcillin, can inactivate MRSA's four high-binding-affinity PBPs [66].

In the present study, *Streptococcus pyogenes* isolates were 90% susceptible to azithromycin, 60% to tetracycline, 66.7 % to erythromycin, and 90% to clindamycin. The finding is similar as compared with the report found in Nepal [67], and Ethiopia, Hawassa [55]. Mechanisms of antimicrobial resistance in *Streptococcus pyogenes* are attributable to the mef (A) gene, which was first identified as the resistance determinant responsible for macrolide type M resistance.

In the current study, *Haemophilus influenzae* and *Klebsiella species* isolates showed higher level of resistance to 94.1% tetracycline, 88.2% ampicillin,76.5% amoxacillin-clauvlic acid and 73.5% cotrimoxazole and chloramphenicol. This could be due to the overuse of these drugs for many years, the expression of extended-spectrum β-lactamases, which develop resist against penicillin, cephalosporins, and monobactams, and the expression of carbapenems, which provides resistance against those β-lactams including carbapenems. The capsule, production of biofilm, efflux pumps and production of polysaccharide matrix that coats the cell can limit the penetration of certain agents [68, 69]. On the other hand, lower resistance was observed against gentamicin, amikacin, ciprofloxacin and meropenem which is suggestive of a possible drug of choice for such infections.

In the present study participants who had large family-size showed statistically significant association with the occurrence of with nasopharyngeal bacteria carriage [AOR=2.17, 95%CI 1.24–3.81, P=0.006], which is comparable with studies reported in Indian (P = 0.03)[70], Iran (p=0.044) [62], Ethiopia in Addis Ababa (p=0.006) [7], and Gondar (P= 0.031) [71]. The other factor in the present study that has statically significant association was numbers of rooms (1-2) per house with [AOR=5.88, 95%CI: 1.26-27.57, P=0.024]. This might be due to the fact that an increase in the number of family members leads to greater sharing of aerosol droplets which causes more spread of bacteria among family members. This finally could have a role in bacteria carriage for healthy individuals.

Another independently associated factor statically associated with nasopharyngeal bacteria carriage was children with a history of hospitalization [AOR=4.08, 95%CI 1.45–1.53, P=0.008]. This is in agreement with the studies conducted in Ethiopia, Hawassa (p=0.00) [55], Gondar (P= 0.031) [71]. Common type of nosocomial infection involves invasive devices and procedures (urinary catheters, central lines, mechanical ventilation, or surgery) which might be an important factor for the transmission of respiratory infections and it could have a role in bacteria carriage for healthy individuals [72].

The other factor that contributed to nasopharyngeal bacteria carriage was passive smoking [AOR = 4.87, 95%CI 1.49–15.97, P = 0.009]. This finding indicated that nasopharyngeal bacteria carriage were 4.87 times more likely to be developed among children who had passive smoking habits. This is also in agreement with a study done in Iran (p = 0.045) [62], Ethiopia, Gondar (P = 0.004) [55]. The potential reason for this association is that active and passive smoking damages the upper layer of the mucosal surface of the respiratory tract, which favors the carriage of bacteria in the nasopharynx.

The number of students in the classroom [AOR=2.35,95%CI 1.37–4.02, P=0.002] was a significant risk factor for nasopharyngeal bacteria carriage. Children with greater than 25 classmates per class room had 2.35 times more risk to nasal bacteria carriage. This result was consistent with the study conducted in Ethiopia, Gondar (P=0.048) [55], and Jimma (p=0.016) [49]. This showed that the increased in the number of students per classroom, the more likely the nasal carriage was observed among school children. The possible reason might be because a high number of students in one classroom makes them more frequent contact with each other, overcrowding, and greater sharing of nasal flora which causes the more spread of the bacteria.

## Conclusion and recommendation

From 384 samples collected from primary school children, the overall bacterial carriage was 35.7%. Among them, predominant isolates were* Staphylococcus aureus* 53.3% (73/137). The overall MDR of isolated bacteria was 62.03%. Gram-positive bacteria showed more than 50% of resistance for tetracycline 87 (84.5%), trimethoprim-sulfamethoxazole 69 (74.2%), doxycycline 80 (86%), and chloramphenicol 70 (68%). Similarly, most of the isolated Gram-negative bacteria showed resistance of 94.1% for tetracycline, 88.2% for ampicillin, and 76.5% for amoxacillin-clavulinic acid. However, the most effective drugs were amikacin and meropenem. Children who had a history of hospitalization, family size of > 5 members, passive smoking, greater than twenty-five students per classroom, and numbers of rooms per house were identified as associated risk factors for nasopharyngeal bacteria carriage. Health education programs should be conducted on the factors for nasopharyngeal infections at a large scale for lower-level education students. The number of students per classroom in each grade level should be minimized. Further relevant studies need to be conducted among asymptomatic children of different levels other than primary school, such as kindergarten and daycare children, and in different geographical locations to follow the carriage and species identification. The molecular analysis is recommended to confirming the presence of a resistant gene on MRSA carriage.

## Limitation of the study

Species investigation was not performed for CoNS due to financial issue and shortage of reagents and materials. Moreover, molecular characterization of the isolated bacterial agents, detection of virulence and antimicrobial resistance genes was not performed. Similarly, the correlation between the phenotypic and genotypic MDR was not performed. Moreover, it was facility-based cross-sectional study conducted at a point in time. Thus, exposure and outcome were simultaneously assessed; there is generally limited capacity to assess causality between exposure and outcome.

## Data Availability

Data supporting the conclusions of this article are within the manuscript.

## Abbreviations

AMR: Antimicrobial resistance
AOR: Adjusted odds ratio
ATCC: American type cell culture
CLSI: Clinical Laboratory Standard Institute
BAP: Blood agar plate
CAP: Chocolate agar plate
CI: Confidence interval
CoNS: Coagulase negative *Staphylococcus*
COR: Crude odds ratio
MDR: Multidrug resistance
MHA: Muller Hilton Agar
SOPs: Standard operating procedures
SPSS: Statistical package for social science
AS: Abdurahaman Seid
AGS: Agumas Shibabaw
BS: Bekele Sharew
MAB: Melaku Ashagrie Belete
MT: Mihret Tilahun
CB: Chernet Belayhun
WD: Wondmagegn Demsiss
